# Single-cell RNA sequencing identifies inherent abnormalities of adipose-derived stem cells from nonlesional sites of patients with localized scleroderma

**DOI:** 10.1186/s11658-024-00635-0

**Published:** 2024-08-30

**Authors:** Xuanyu Liu, Zhujun Li, Hayson Chenyu Wang, Meng Yuan, Jie Chen, Jiuzuo Huang, Nanze Yu, Zhou Zhou, Xiao Long

**Affiliations:** 1 State Key Laboratory of Cardiovascular Disease, Beijing Key Laboratory for Molecular Diagnostics of Cardiovascular Diseases, Center of Laboratory Medicine, Fuwai Hospital, National Center for Cardiovascular Diseases, Chinese Academy of Medical Sciences and Peking Union Medical College, Beijing, 100037 China; 2 Department of Plastic Surgery, State Key Laboratory of Complex Severe and Rare Diseases, Peking Union Medical College Hospital, Chinese Academy of Medical Science and Peking Union Medical College, Beijing, 100730 China; 3 Department of Plastic and Reconstructive Surgery, Shanghai Ninth People’s Hospital, Shanghai Jiao Tong University School of Medicine, Shanghai, 200011 China

**Keywords:** Localized scleroderma, Adipose-derived stem cells, Stromal vascular fraction, CD55, Single-cell RNA sequencing

## Abstract

**Background:**

Localized scleroderma (LoS) is an autoimmune disorder that primarily affects the skin, and is often treated with autologous fat grafting (AFG). Nevertheless, the retention rate of AFG in patients with LoS is typically low. We hypothesize that the low retention rate may be partially attributed to the inherent abnormalities of adipose-derived stem cells (ASCs) from nonlesional sites of patients with LoS.

**Methods:**

We performed a comparative analysis of the single-cell transcriptome of the SVF from nonlesional sites of patients with LoS and healthy donors, including cellular compositional analysis, differential expression analysis, and high-dimensional weighted gene coexpression network analysis. Experimental validation with fluorescence-activated cell sorting and bleomycin-induced skin fibrosis mice models were conducted.

**Results:**

We found a significant reduction in the relative proportion of CD55^high^ interstitial progenitors in ASCs under the condition of LoS. Differential expression analysis revealed inherent abnormalities of ASCs from patients with LoS, including enhanced fibrogenesis, reduced anti-inflammatory properties, and increased oxidative stress. Compared with CD55^low^ ASCs, CD55^high^ ASCs expressed significantly higher levels of secreted protein genes that had functions related to anti-inflammation and tissue regeneration (such as *CD55*, *MFAP5*, and *METRNL*). Meanwhile, CD55^high^ ASCs expressed significantly lower levels of secreted protein genes that promote inflammation, such as chemokine and complement protein genes. Furthermore, we provided in vivo experimental evidence that CD55^high^ ASCs had superior treatment efficacy compared with CD55^low^ ASCs in bleomycin-induced skin fibrosis mice models.

**Conclusions:**

We found that the low retention rate of AFG may be partially ascribed to the reduced pool of interstitial progenitor cells (CD55^high^) present within the ASC population in patients with LoS. We demonstrated the potential for improving the efficacy of AFG in the treatment of LoS by restoring the pool of interstitial progenitors within ASCs. Our study has significant implications for the field of translational regenerative medicine.

**Supplementary Information:**

The online version contains supplementary material available at 10.1186/s11658-024-00635-0.

## Introduction

Localized scleroderma (LoS) is an autoimmune disorder that primarily affects the skin [1]. Although the etiology of LoS is not well understood, it is believed to be related to an overactive immune system [2]. LoS is characterized by microvessel damage, dermal fibrosis, and subcutaneous adipose tissue atrophy, leading to thickening and hardening of the skin [2]. LoS lesions on the face can significantly impact a patient’s appearance [1]. Unfortunately, there is currently no cure for this condition. However, treatments, such as topical creams, oral medications, light therapy, and surgery, can help relieve symptoms and delay disease progression [1, 3].

Among these therapies, surgery remains the only viable option to correct contour deformities caused by LoS. Autologous fat grafting (AFG) is one of the commonly used surgical methods that involves injecting a patient’s own fat from nonlesional areas into lesion sites [3]. In addition to providing bulk and support, AFG also alleviates immune responses and relieves fibrosis primarily through the adipose-derived stem cells (ASCs) within adipose tissue [4]. ASCs belong to mesenchymal stromal cells (MSCs) that possess immunomodulatory, pro-angiogenic, anti-fibrotic, and multilineage differentiation properties [5–7]. Recent studies have demonstrated the potential of ASCs as a promising new treatment option for scleroderma.

Nonetheless, the retention rate of autologous fat grafts in patients with LoS was usually low, often necessitating multiple rounds of fat grafting [3, 4, 8]. Previous studies suggested dysfunctions of ASCs under conditions of scleroderma. For example, compared with ASCs from healthy individuals, ASCs from patients with progressive hemifacial atrophy (PHA), a subtype of LoS, exhibited reduced ability to promote fat graft survival [9]. Studies on other forms of scleroderma, for example, systemic scleroderma (SSc), have also provided evidence of inherent abnormalities in differentiation capacity and biological functions of MSCs from patients compared with those from healthy donors [10]. Therefore, we hypothesize that the low retention rate of autologous fat grafts in patients with LoS may be partially attributed to the inherent abnormalities of ASCs from nonlesional sites of patients with LoS, although LoS is commonly regarded as a localized disease without systemic involvement. To better understand the factors contributing to the low retention rate of autologous fat grafts from patients with LoS, a systematic comparison of the transcriptome between autologous fat or ASCs from patients and healthy donors is needed.

Single-cell RNA sequencing (scRNA-seq) is a powerful tool that allows for cell-type-resolved transcriptomic analysis in complex tissues with heterogeneous cellular states. Previous studies have successfully applied single-cell transcriptomic analysis to investigate the heterogeneity of stromal vascular fraction (SVF), which consists of nonadipocyte cells in adipose tissue, under both healthy and diseased conditions [11, 12]. In this study, we performed a comparative analysis of the single-cell transcriptome of the SVF from nonlesional sites of patients with LoS and healthy donors. We found a significant reduction in the relative proportion of *CD55*^high^ interstitial progenitor cells in ASCs under the condition of LoS. Differential expression analysis confirmed the hypothesis of inherent abnormalities of ASCs from patients with LoS, which were found to exhibit enhanced fibrogenesis, reduced anti-inflammatory properties, and increased oxidative stress. The functional abnormalities in ASCs and the low retention rate of autologous fat may be partially ascribed to the reduced pool of interstitial progenitor cells (*CD55*^high^) present within the ASC population. Compared with *CD55*^low^ ASCs, *CD55*^high^ ASCs expressed significantly higher levels of secreted protein genes that had functions related to anti-inflammation and tissue regeneration (such as *CD55*, *MFAP5*, and *METRNL*). Meanwhile, *CD55*^high^ ASCs expressed significantly lower levels of secreted protein genes that promote inflammation, such as chemokine and complement protein genes. Furthermore, we provided in vivo experimental evidence that *CD55*^high^ ASCs had superior treatment efficacy compared with *CD55*^low^ ASCs in bleomycin-induced skin fibrosis mice models. Our study demonstrated the inherent abnormalities of ASCs from nonlesional sites of patients with LoS and uncovered superior treatment efficacy of *CD55*^high^ ASCs. These findings have significant implications for the field of translational regenerative medicine.

## Materials and methods

### Subject enrollment

The inclusion criteria for the LoS patient group were as follows: (i) females aged 18–60 years old, (ii) newly diagnosed patients with LoS who have not received steroid or fat grafting treatment, and (iii) presence of facial contour deformities and planning to undergo AFG surgical treatment at the department of plastic surgery in Peking Union Medical College Hospital. The exclusion criteria included: (i) suffering from cancer or infectious diseases and (ii) suffering from other skin-affecting diseases. As controls, healthy individuals who underwent liposuction surgeries for cosmetic purposes were also recruited. The two groups were required to be matched in terms of sex, age, body mass index (BMI), and ethnicity.

### Liposuction and sample collection

Subcutaneous adipose tissue specimens were collected from the thighs of healthy donors or the nonlesional thighs of patients with linear scleroderma lesions on the face. Both groups underwent the same liposuction procedures performed by the same plastic surgery division. Before liposuction, general anesthesia was administered. To prepare for liposuction, a tumescent solution was injected into the thigh. The solution contained 0.025% lidocaine and 1:1,000,000 epinephrine in 1000 ml of fluid. Adequate infiltration was confirmed by tissue blanching and moderate tension. Liposuction was performed using 3 mm blunt-tip cannulas and 20 ml syringes in the deep fat layer. The syringes were then left undisturbed for 10 min to allow separation of the adipose tissue from the tumescent fluid. The harvested adipose tissues were transferred to 50 ml sterile centrifuge tubes containing 20 ml of Dulbecco’s modified Eagle’s medium (10569044, Gibco) cell culture medium and kept on ice during transportation.

### SVF isolation

To prepare the fat tissue for SVF isolation, it underwent several washes with Hank’s balanced salt solution (14025126, Gibco). Thereafter, it was digested at 37 °C for 30 min using 0.15% collagenase I (17100017, Gibco) supplemented with 4% penicillin–streptomycin (15140122, Gibco). Following centrifugation at 1000 rpm for 10 min, the resulting cell pellet was resuspended in high-glucose Dulbecco’s modified Eagle’s medium (10569044, Gibco) containing 10% fetal bovine serum (10099141, Gibco). The suspension was then filtered through a 100-μm strainer and centrifuged at 1500 rpm for 5 min. Subsequently, the obtained cell suspension was resuspended in Hank’s balanced salt solution, and red blood cell lysis buffer was added at room temperature for 5 min to eliminate red blood cells. Another round of centrifugation was performed, and the cell pellet was resuspended in Hank’s balanced salt solution with 0.04% bovine serum albumin (A1933-5G, Sigma) before being filtered through a 40-μm strainer. Finally, the cells were centrifuged and resuspended in Dulbecco’s phosphate buffered saline without Ca^2+^ and Mg^2+^ (14190144, Gibco). To assess cell concentration and viability, the resulting single-cell suspension was incubated with an equal volume of AOPI Staining Solution (Logos Biosystems) and analyzed using a LUNA-FL Fluorescence Cell Counter (Logos Biosystems).

### Single-cell transcriptomic sequencing

The Chromium Single-Cell 3′ Reagent Kit v3 (10 × Genomics, USA) was used for various steps, including single-cell gel bead-in-emulsion (GEM) making, post-GEM-RT cleanup, barcoding, cDNA amplification, and cDNA library construction, following the manufacturer’s protocol. Library sequencing was carried out using the NovaSeq 6000 system (Illumina, USA).

### Preprocessing of the scRNA-seq data

The sample demultiplexing, barcode processing, and unique molecular identifier (UMI) counting were performed using the official software Cell Ranger (v3.0.2). The FASTQ data was demultiplexed using “cellranger mkfastq,” and the resulting gene-barcode matrix for each library was generated with the “cellranger count” pipeline. To reduce noise, genes expressed in fewer than three cells were excluded, and potential poor-quality cells were removed based on their expressed gene number, UMI count sum, and percentage of mitochondrial genes. Scrublet (v0.2.3) was used to predict and remove doublets. The quality control thresholds for each sample are provided in Additional file 1: Table S1. Cells with enriched hemoglobin gene expression were identified and removed as potential red blood cells. The UMI counts for each cell were normalized to 10,000 and log-transformed. For each sample, the “FindVariableFeatures” function of Seurat (v3.1.0) was applied to select 2000 features (genes). The canonical correlation analysis in Seurat was used to integrate the datasets and correct potential batch effects under default settings. Linear regression was used to regress out variation from mitochondrial gene proportion, S phase score, G2M phase score, and UMI count. Principal component analysis (PCA) was used for linear dimensional reduction, with the first 30 principal components used to construct the neighborhood graph of the cells (Fig. S1). The uniform manifold approximation and projection (UMAP) algorithm was used to embed the graph in a two-dimensional space. The cells were then clustered using Louvain clustering (resolution = 0.6) implemented in Seurat.

### Cellular annotation through reference mapping

Cellular annotation was performed using the data transfer workflow of Seurat with the scRNA-seq data of SVF that has previously been published by our lab [11] as a reference. Briefly, the function FindTransferAnchors was used to find anchors between two datasets. Then, the function TransferData was used to classify the query cells based on reference data.

### Differential compositional testing

To detect statistically credible alterations in cellular composition derived from the single-cell dataset, we used a Bayesian approach implemented in scCODA (v0.1.9) [13] (reference_cell_type = “automatic,” Hamiltonian Monte Carlo sampling method with default settings).

### Differential expression analysis

The identification of differentially expressed genes (DEGs) between groups was performed using the FindMarkers function of Seurat. The test utilized for this analysis was the likelihood-ratio test with the “bimod” option. Genes were considered significantly different in expression between the two groups if they showed an absolute log2-fold change exceeding 0.25 and an adjusted *P*-value lower than 0.05.

### Gene set enrichment analysis

We first ranked all the expressed genes using the Signal2Noise method, which normalized the mean difference between LoS and the control (CTRL) with the standard deviation. The resulting ranked list of genes was subsequently imported into the GSEA software (version 4.0.1). A statistically significant threshold was established with an FDR *q* value below 0.05. For the analysis, the precompiled REACTOME pathways from MSigDB (version 7.0) were utilized. The visualization of the results was accomplished using the EnrichmentMap plugin incorporated in Cytoscape (version 3.7.0).

### High-dimensional weighted gene coexpression network analysis

The high-dimensional weighted gene coexpression network analysis (hdWGCNA) was conducted using the hdWGCNA R package (v0.2.04) [14] with default parameters. This package has been specifically designed to analyze high-dimensional scRNA-seq data. Within each module, the hub genes were determined by identifying the top 25 genes ranked by eigengene-based connectivity (kME).

### Intercellular communication analysis

The CellChat (v1.6.1) package [15] was utilized to deduce interactions between cell types within each group and detect changes in intercellular communication through comparative analysis. Briefly, the approach involved identifying overexpressed ligands or receptors for each cell type and quantifying potential interactions between any two cell types using a communication probability value. Significant interactions (*P*-value < 0.05) were identified by employing a permutation test, where cell type labels were randomly permuted, and the communication probability was recalculated. The use of pattern recognition techniques helped detect prominent incoming and outgoing signal patterns for each cell type in each condition. Network centrality analysis was used to infer the main sources and targets of the signaling network for a particular pathway. The identification of significantly altered signaling pathways was achieved through a comparative analysis of the overall information flow within each signaling pathway by assessing the sum of communication probability among all pairs of cell types in the inferred network.

### Fluorescence-activated cell sorting

To eliminate dead cells, freshly isolated SVF was suspended in phosphate-buffered saline (PBS) containing 1:1000 Zombie NIR dye (423105, BioLegend) and incubated at room temperature in the dark for 15 min. After washing with cell staining buffer (420201, BioLegend), the cells were resuspended and incubated with cellular surface antibodies, including PE anti-human CD140a (PDGFRα) antibody (323506, BioLegend) and APC anti-human CD55 antibody (311312, BioLegend), at room temperature in the dark for 30 min. The cells were washed twice with cell staining buffer by centrifugation at 350×*g* for 5 min. Flow cytometry data were obtained using the LSRFortessa flow cytometer (BD Biosciences) and analyzed using the FlowJo software (BD Biosciences). The CD55^high^ ASCs were finally sorted using fluorescence-activated cell sorting (FACS) on Beckman Moflo Astrios EQ (Beckman-Coulter).

### Bleomycin-induced mice models for skin fibrosis and ASC treatment

To compare the anti-fibrotic effects of CD55^high^ and CD55^low^ ASCs in patients with LoS, we established a mouse model using bleomycin (BLM) induction to mimic the skin fibrosis and subcutaneous adipose tissue loss observed in human patients. To avoid potential immune rejection from xenografts, partially immunodeficient Balb/c nude mice were used since the ASCs used for treatment were isolated from healthy human donors. A total of 20 female Balb/c nude mice aged 6 weeks were randomly assigned to one of four groups: PBS control, model control, CD55^high^ ASC subcutaneous injection, and CD55^low^ ASC injection groups. During model construction, all mice received daily subcutaneous injections of 20 μg of BLM (HY-17565, MedChemExpress, NJ, USA) dissolved in 100 μl of PBS in the lower back for 30 days, except for the PBS control group, which received the same volume of PBS without BLM. The successful establishment of skin fibrosis models was confirmed by skin appearance and histological staining. A day after the last injection of BLM, freshly isolated CD55^high^ ASCs and CD55^low^ ASCs were subcutaneously injected into the lower back of the mice at a concentration of 1 × 10^4^ cells per animal, suspended in 100 μl of PBS. The PBS control and model control groups received 100 μl of PBS without ASCs at the same site. After 30 days, the mice were sacrificed, and skin samples from the lower back were collected for further experiments. All 20 mice survived the treatments and were included in further analysis.

### In vivo fluorescence imaging

Three mice were injected subcutaneously with bleomycin to induce a scleroderma skin fibrosis model, as described above. ASCs at a concentration of 5 × 10^7^ cells/mL were labeled with 2.5 µmol/L DiD by incubating them at 37 °C for 20 min. The DiD-labeled ASCs were then injected subcutaneously into the lower back of the mice at a dose of 5 × 10^6^ cells per animal. Fluorescence signal intensity was quantified using a PerkinElmer IVIS Lumina III system at days 0, 2, 7, 14, 21, 28, 35, and 46. Relative signal intensity was determined by comparing the fluorescence signal at each time point with that at day 0.

### Paraffin section preparation

Skin samples from the mice were fixed overnight at 4 ℃ in a 4% paraformaldehyde solution (G1101, Servicebio, Wuhan, China) and subsequently embedded in paraffin. Deparaffinization of 5-μm sections was carried out using xylene, followed by rehydration in alcohol and rinsing in distilled water before further staining.

### Hematoxylin and eosin staining

The rehydrated sections were stained using the hematoxylin and eosin (HE) staining kit (G1005, Servicebio, Wuhan, China) following the manufacturer’s protocol. After staining, the sections were dehydrated and covered with neutral balsam mounting medium (10004160, Sinopharm, Shanghai, China). Scanning of the stained sections was performed using the Pannoramic SCAN (3DHISTECH) system. The thickness of the epidermis, dermis, and subcutaneous adipose tissue was measured from six random regions per section using ImageJ software.

### Masson’s trichrome staining

The rehydrated sections were stained using a Masson’s trichrome staining kit (G1006, Servicebio, Wuhan, China) following the manufacturer’s protocol. After staining, the sections were scanned using the Pannoramic SCAN (3DHISTECH) system. The blue-stained collagen area was quantified using ImageJ software from three random regions per section. The collagen volume fraction (CVF) was then calculated using the formula: CVF (%) = collagen area / full area × 100%.

### Immunohistochemistry staining

The rehydrated sections were subjected to antigen retrieval by heating in sodium citrate buffer (pH 6.0). To eliminate endogenous peroxidase, 3% H_2_O_2_ was used. Subsequently, the sections were blocked with 3% bovine serum albumin (GC305010, Servicebio, Wuhan, China) for 30 min and incubated with primary antibodies overnight at 4℃. After washing off unbound primary antibodies, horseradish peroxidase (HRP)-conjugated secondary antibodies were applied, followed by DAB treatment and hematoxylin staining. The primary antibodies used included TGFβ1 (GB14154-50, Servicebio, Wuhan, China), Adiponectin (21613-1-AP, Proteintech, Wuhan, China), and PPARγ (16643-1-AP, Proteintech, Wuhan, China). The sections were dehydrated, covered with mounting medium, and scanned. Integrated optical density (IOD) values were measured from six random regions per section using ImageJ.

### Western blotting

Mice skin samples were lysed using cold RIPA buffer (P0013, Beyotime Biotechnology, Shanghai, China) supplemented with protease/phosphatase inhibitors (P1045, Beyotime Biotechnology, Shanghai, China) using a tissue grinder. The lysed samples were then centrifuged at 12,000*g* for 10 min to remove debris. Protein concentrations were determined using the Thermo Scientific Pierce BCA Kit (23225, Thermo Scientific). For western blotting analysis, 20–40 μg of protein per sample was loaded onto a 10% sodium dodecyl-sulfate (SDS)–polyacrylamide gel and separated by electrophoresis. The proteins were then transferred onto 0.45 μm PVDF membranes and blocked with 5% nonfat milk (P0216, Beyotime Biotechnology, Shanghai, China) in Tris-buffered saline with tween-20 (TBST, ST671, Beyotime) for 1 h at room temperature. Primary antibodies against collagen I (1:2000, ab260043, Abcam) and α-SMA (1:2000, 14976082, Invitrogen) were incubated overnight at 4℃. Glyceraldehyde 3-phosphate dehydrogenase (GAPDH; 1:2000, 2118L, Cell Signaling Technology) was used as a control. After washing off the unbound antibodies with TBST, the membranes were incubated with secondary antibodies conjugated with HRP for 1 h at room temperature. The protein bands were detected using enhanced chemiluminescence (ECL; P0018A, Beyotime Biotechnology, Shanghai, China), and the intensity of the bands was quantified using ImageJ.

### Statistical analysis

Statistical analyses were performed using GraphPad Prism (version 9). Two-tailed Wilcoxon rank-sum test or Student’s *t*-test was used to compare two groups of data, while one-way analysis of variance (ANOVA) was used to analyze data among multiple groups. Following ANOVA, a post hoc test was performed using Tukey’s honest significant difference (HSD). A *P*-value of less than 0.05 was considered statistically significant, denoted by *, **, ***, and **** to indicate *P*-values of less than 0.05, 0.01, 0.001, and 0.0001, respectively.

## Results

### Single-cell transcriptomic sequencing of subcutaneous adipose tissues from nonlesional sites of patients with LoS

**Fig. 1:**
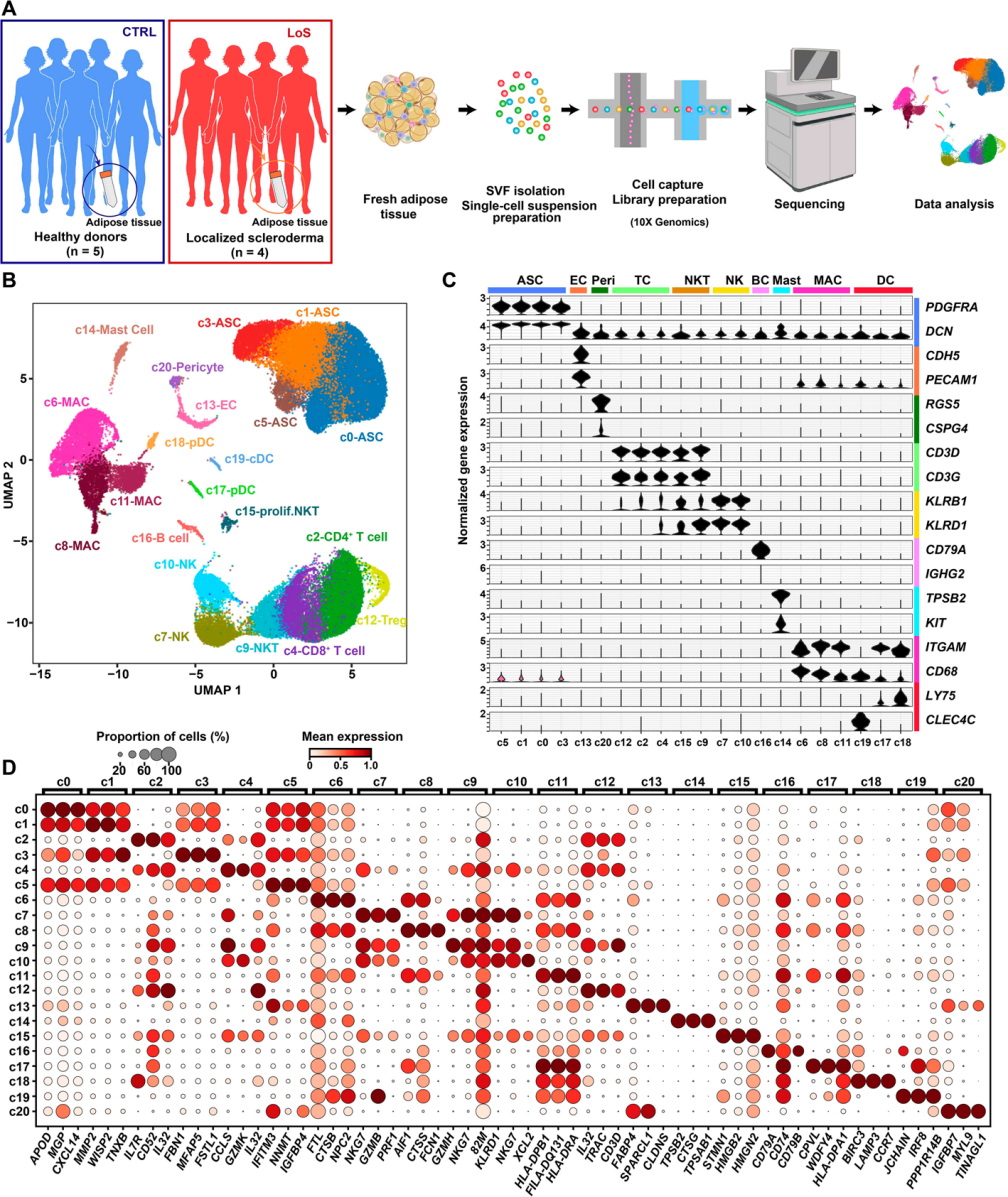
Cellular heterogeneity of subcutaneous adipose tissues from nonlesional sites of patients with LoS. **A** Schematic representation of the experimental procedure. During surgery, liposuction specimens were obtained from nonlesional sites of the thighs of patients with localized scleroderma (LoS) (*n* = 4) and healthy donors (*n* = 5). The stromal vascular fraction (SVF) was then isolated from the collected subcutaneous adipose tissue, and single-cell RNA sequencing (scRNA-seq) was conducted on each sample individually. **B** UMAP plot showing the distribution of cellular clusters of all sequenced cells. Each dot denotes a cell and is color-coded according to its respective cellular cluster. **C** Violin plot showing the normalized expression of canonical markers for each cell type. **D** Dot plot showing the expression of representative molecular signatures for each cluster. The size of the circles represents the proportion of cells expressing the gene, while the intensity of the color reflects the level of expression. *ASC* adipose-derived stem cell, *cDC* conventional dendritic cell, *CTRL* control, *EC* endothelial cell, *LoS* localized scleroderma, *MAC* macrophage, *NK* natural killer cell, *NKT* natural killer T cell, *prolif.NKT* proliferative nature killer T cell, *pDC* plasmacytoid dendritic cell

Liposuction specimens were obtained from nonlesional sites of the thighs of patients with LoS (*n* = 4) and healthy donors (CTRL; *n* = 5). The two groups were matched in terms of sex, age, BMI, and ethnicity (Additional file 1: Table S1). The SVF was then isolated from the collected adipose tissue, and scRNA-seq was conducted on each sample individually (Fig. 1A). After quality filtering, 58,015 high-quality cells were obtained. The scRNA-seq data of four samples of the CTRL group has been previously published by our lab and a well-annotated single-cell transcriptomic atlas of SVF has been generated [12]. To annotate the cells based on the previous annotation, we applied Seurat’s data transfer workflow with the previously published dataset [12] as a reference. The data transfer result showed that our dataset encompassed 21 cellular clusters that could be assigned to 10 cell types (Fig. 1B–D and Additional file 2: Table S2), including ASCs (marked by *PDGFRA* and *DCN*), endothelial cells (marked by *CDH5* and *PECAM1*), pericytes (marked by *RGS5* and *CSPG4*), macrophages (marked by *ITGAM* and *CD68*), mast cells (marked by *TPSB2* and *KIT*), dendritic cells (marked by *LY75* and *CLEC4C*), T cells (marked by *CD3D* and *CD3G*), B cells (marked by *CD79A* and *IGHG2*), natural killer (NK) cells (marked by *KLRB1* and *KLRD1*), and natural killer T (NKT) cells (expressing both NK and T cell markers). The ASC lineage represents the largest nonimmune cell type in SVF and includes four subclusters: c0, c1, c3, and c5. On the basis of the reference annotation, c0 (*GGT5*^high^) represents committed preadipocytes; c3 (*CD55*/*DPP4*^high^) represents multipotent interstitial progenitor cells; c1 (*MMP2*^high^) represents intermediate states between interstitial progenitor and committed preadipocytes; and c5 (expressing high levels of metallothionein genes) represents a stress-responsive subpopulation. The macrophages encompassed three subclusters: c6 (*LYVE1*^high^) represents resident-like macrophages with an M2 anti-inflammatory phenotype, c8 (*IL1B* high) proinflammatory macrophages, and c11 (expressing high levels of MHC class II genes) represents antigen-presenting macrophages.

### Cellular compositional analysis revealed a significant reduction in the relative proportion of ***CD55***^high^ interstitial progenitor cells in ASCs under the condition of LoS

**Fig. 2:**
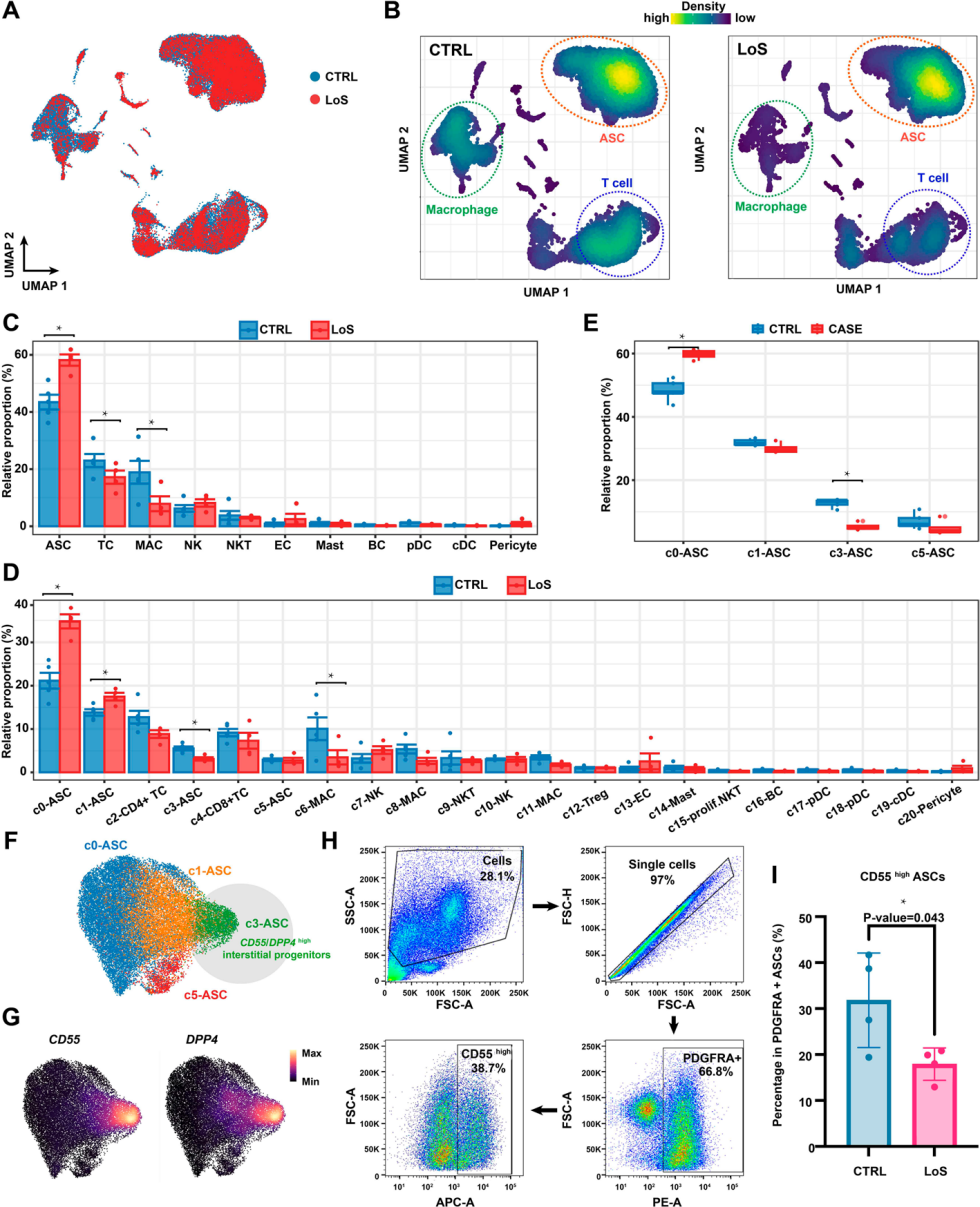
Cellular compositional analysis revealed a reduced relative proportion of *CD55*^high^ interstitial progenitor cells in ASCs under the condition of LoS. **A** UMAP plot showing the cellular distribution of each group. **B** UMAP plots showing the kernel density of cellular distribution for each group. **C** Bar plot showing the relative proportion of each cell type in the SVF for each group. **D** Bar plot showing the relative proportion of each subcluster in the SVF for each group. In C and D, the data are presented as the mean ± SEM (CTRL: *n* = 5; LoS: *n* = 4). **E** Box plot showing the relative proportion of each subcluster in ASCs for each group. In C–E, *: statistically credible change in cellular composition determined by a Bayesian method implemented in scCODA. **F** UMAP plot showing the four subclusters of ASCs. **G** Expression of markers for ASC interstitial progenitor cells (*CD55* or *DPP4*). The visualization was enhanced by using the R package Nebulosa to recover the signal from dropped-out features. **H** Gating strategy for sorting CD55^high^ ASCs from SVF using FACS. **I** FASC analysis confirmed the significantly reduced relative proportion of CD55^high^ interstitial progenitor cells in ASCs under the condition of LoS. The data are presented as the mean ± SEM (*n* = 4 for each group). * *P*-value < 0.05, Student’s *t*-test. *CTRL* control, *LoS* localized scleroderma

Next, we sought to identify alterations in the cellular composition of the SVF under the condition of LoS. From the UMAP plot (Fig. 2A), we observed a contraction of macrophages under the condition of LoS. However, this form of visualization suffered from the overplotting problem. To avoid this, we calculated the kernel density of the cells for each group (Fig. 2B). From the kernel density plot, we observed contractions of both macrophages and T cells. The peak of the kernel density altered in ASCs, implying a shift in transcriptomic status under the pathological condition. Then, through a quantitative comparison of the relative proportions of cell types between groups, we found a statistically significant expansion of ASCs and contractions of macrophages and T cells (Fig. 2C, Fig. S2A; a Bayesian method implemented in scCODA). Furthermore, we identified statistically significant expansions of the subclusters c0-ASC and c1-ASC in SVF, and significant contractions of the subclusters c3-ASC and c6-MAC (Fig. 2D, Fig. S2B). When only considering the relative proportion in ASCs (Fig. 2E), we again detected a significant expansion of the subcluster c0-ASC (*GGT5*^high^ committed preadipocytes) and a significant contraction of the cluster c3-ASC (*CD55*^high^ interstitial progenitor cells). A previous study reported that reduced multipotent interstitial progenitor cell pool (*CD55/DPP4*^high^) in ASCs may contribute to the pathologic remodeling in obesity^10^. Consistent with the previous report, we also observed the presence of *CD55*/*DPP4*^high^ interstitial progenitor cells in our dataset and a significant reduction in the pathological state of LoS (Fig. 2F, G; Fig. S3). Through FACS, we confirmed the significantly reduced relative proportion of CD55^high^ interstitial progenitor cells (c3-ASC) in ASCs under the condition of LoS (Fig. 2H, I).

### Cell-type-resolved differential expression analysis revealed dysregulated pathways in ASCs from nonlesional sites of patients with LoS

**Fig. 3:**
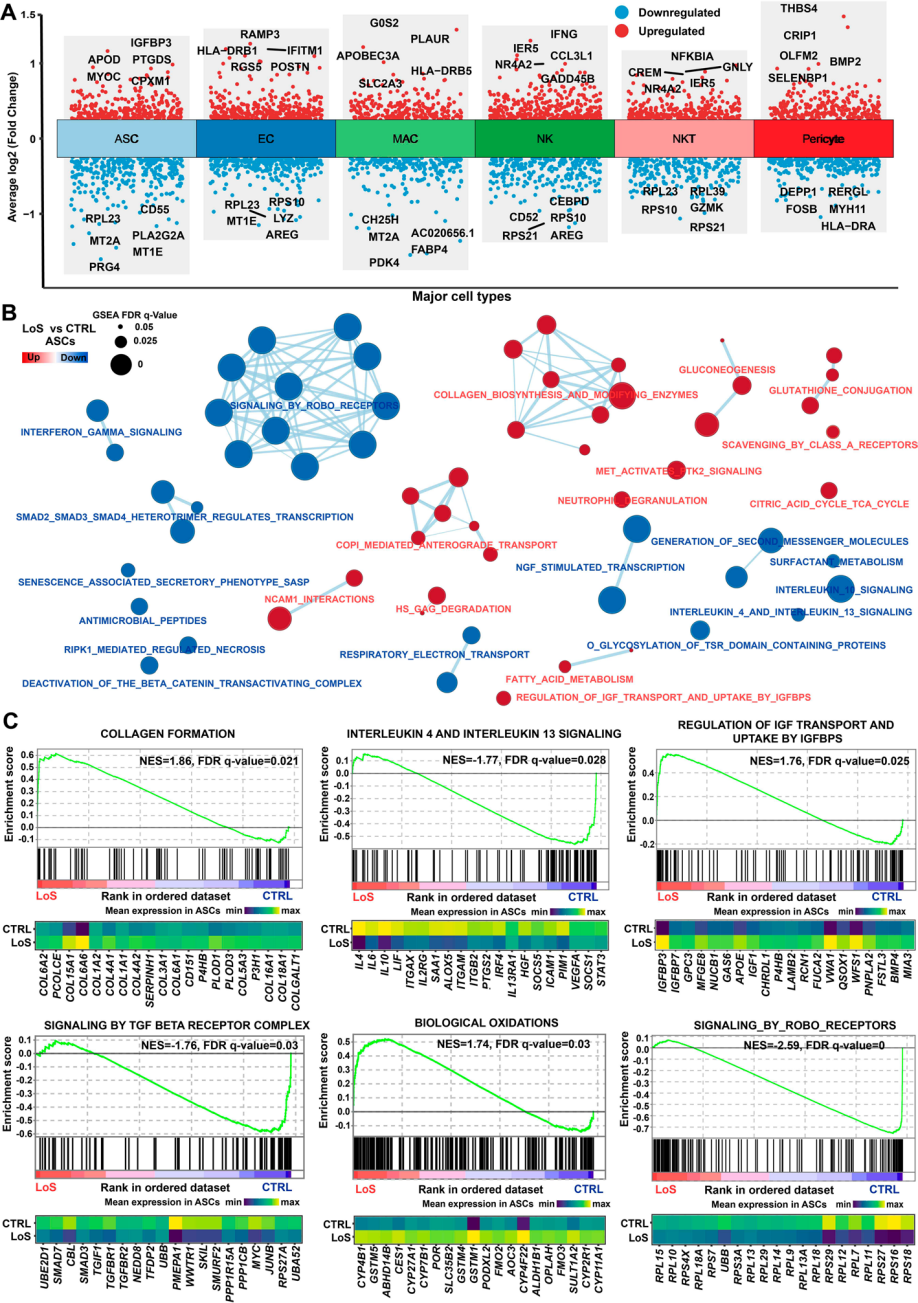
Cell-type-resolved expression differences between the SVF from nonlesional sites of patients with LoS and healthy donors. **A** Differentially expressed genes that were identified in each major cell type between LoS and CTRL. The likelihood-ratio test implemented in Seurat was applied. A statistical threshold was set to an absolute log2-fold change > 0.25 and an adjusted *P*-value < 0.05. **B** Dysregulated pathways in ASCs from nonlesional sites of patients with LoS identified through gene set enrichment analysis (GSEA). A statistically significant threshold was established with an FDR q value < 0.05. **C** Enrichment plots (upper panel) and leading-edge gene expression heatmaps (lower panel; the top 20 genes) for representative pathways dysregulated in ASCs. Normalized enrichment score (NES) was employed to compare analysis outcomes across gene sets. A positive NES indicates that the pathway is upregulated, while a negative score indicates that the pathway is downregulated. The vertical lines depicted in the enrichment plot indicate where the members of the gene set are positioned in the ranked gene list. The subset of genes in the gene set that make the greatest contribution to the enrichment are known as leading-edge genes. The heatmap displays the average expression across cells in each group. *CTRL* control, *FDR* false discovery rate, *LoS* localized scleroderma

Despite the commonly held view that LoS primarily affects the face and remains localized without systemic involvement, our cell-type-resolved differential expression analysis detected hundreds of differentially expressed genes for each major cell type when comparing the SVF from nonlesional sites of patients with LoS with healthy donors (Fig. 3A and Additional file 3: Table S3). This finding suggests functional alterations in cell types of SVF from nonlesional sites of patients with LoS. To gain further insights into the dysregulated pathways, specifically in ASCs from patients with LoS, we conducted gene set enrichment analysis (GSEA; Fig. 3B and Additional file 4: Table S4). ASCs derived from nonlesional sites of patients with LoS demonstrated a notable increase in collagen-related pathways, specifically collagen formation. Several collagen genes, including *COL6A2, COL15A1*, *COL6A6*, *COL1A1*, and *COL3A1*, were found to be upregulated (Fig. 3C). This finding indicated that ASCs from nonlesional sites of patients with LoS exhibited a more synthetic and fibrotic phenotype compared with healthy conditions. Interestingly, the TGFβ signaling pathway, which is implemented in the pathogenesis of tissue fibrosis [16], was unexpectedly found to be downregulated. This finding may suggest a feedback regulation of TGF-β signaling in ASCs from nonlesional sites of patients with LoS. Notably, there was a downregulation of anti-inflammatory signaling pathways, including interleukin 4 and interleukin 13 signaling (Fig. 3C). Cytokines with anti-inflammatory activities, such as *IL4*, *IL6*, and *IL10*, were downregulated (Fig. 3C). These results reflect a reduced anti-inflammatory property of ASCs from patients with LoS. Moreover, the pathway “regulation of IGF transport and uptake by IGFBPS” exhibited significant upregulation, along with the significant upregulation of IGFBP genes such as *IGFBP3* and *IGFBP7*. This indicates the potential involvement of IGFBPs in the functional abnormalities observed in ASCs. Additionally, the pathway associated with biological oxidations showed upregulation, suggesting the presence of oxidative stress in ASCs derived from patients with LoS. Together, our data confirmed the hypothesis of inherent abnormalities of ASCs from patients with LoS, which were found to exhibit enhanced fibrogenesis, reduced anti-inflammatory properties, and increased oxidative stress.

### Gene coexpression modules associated with conditions and cellular states of ASCs

**Fig. 4:**
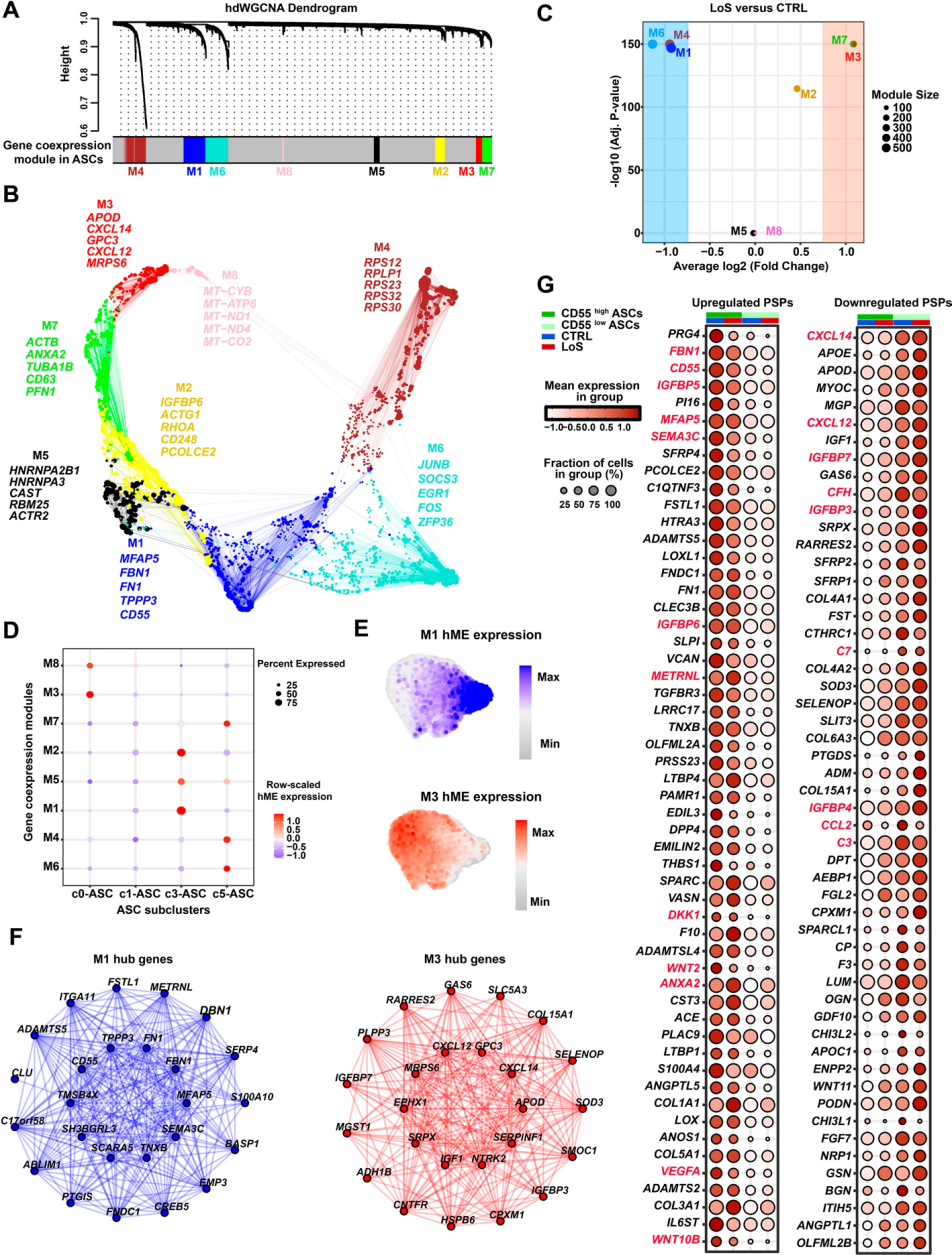
Gene coexpression modules and predicted secreted protein (PSP) genes that were associated with conditions and cellular states in ASCs. **A** Dendrogram showing the gene coexpression modules M1–M8 that were identified in ASCs through high-dimensional weighted gene coexpression network analysis (hdWGCNA). **B** Gene coexpression network of the gene modules. Each dot represents a gene. The hub genes were determined by identifying the top 25 genes ranked by eigengene-based connectivity (kME) in each module. The top five hub genes of each module are shown. **C** Condition-associated gene modules identified by differential module eigengene analysis. Two-tailed Wilcoxon rank sum test. The significance threshold was set to an absolute log2 (fold change) value > 0.75 and a *P*-value adjusted for multiple testing < 0.05. **D** Dot plot showing the expression of harmonized module eigengene (hME) of each module across ASC subclusters. **E** UMAP plots showing the expression distribution of hME for modules M1 and M3. **F** Network plot showing the hub genes of the modules M1 and M3. **G** Dot plots showing the expression patterns of upregulated or downregulated PSP genes in *CD55*^high^ ASCs compared with *CD55*^low^ ASCs. The top 100 differentially expressed PSP genes are shown. Likelihood-ratio test implemented in Seurat. The threshold was set to an absolute log2-fold change > 0.25 and an adjusted *P*-value < 0.05

To gain further insights into the genes associated with the functional abnormalities of ASCs from patients with LoS, we performed high-dimensional weighted gene coexpression network analysis (hdWGCNA). Through this analysis, we identified eight gene coexpression modules in ASCs (Fig. 4A and Additional file 5: Table S5). Hub genes of each module, i.e., the top 25 genes ranked by eigengene-based connectivity, were identified (Fig. 4B). Next, to identify condition-associated modules, differential module eigengene analysis was performed. As shown in Fig. 4C, M1 and M3 were found to be the largest modules that were positively associated with healthy and LoS conditions, respectively. Furthermore, M1 and M3 were found to be primarily expressed in the ASC subclusters c3 (*CD55*^high^ interstitial progenitor cells) and c0 (*GGT5*^high^ committed preadipocytes), respectively (Fig. 4D, E). These findings were in line with the cellular compositional analysis described above, which revealed a contraction of c3 and an expansion of c0 in LoS compared with CTRL (Fig. 2E). Therefore, the gene modules M1 and M3 correspond to the cellular states of interstitial progenitor cells and committed preadipocytes in ASCs, respectively. Modulating the expression of hub genes within these modules has the potential to restore the balance between the two cellular states of ASCs and potentially enhance the efficacy of ASC-mediated treatments. The top hub genes of M1 included *MFAP5*, *FBN1*, *FN1*, *TPPP3*, and *CD55*, while the top hub genes of M3 included *APOD*, *CXCL14*, *GPC3*, *CXCL12*, and *MRPS6* (Fig. 4F).

### Secreted protein genes with differential expression between ***CD55***^high^ and ***CD55***^low^ ASCs

Given the observation that *CD55*^high^ ASCs were contracted in pathological conditions, we hypothesized that CD55^high^ interstitial progenitor cells may have superior treatment efficacy compared with CD55^low^ ASCs, which predominantly consist of committed preadipocytes. To test the hypothesis, we first assessed the expression disparities of genes encoding predicted secreted proteins (PSPs) between *CD55*^high^ and CD55^low^ ASCs, since ASC treatment primarily relies on the actions of secreted proteins. A total of 194 PSP genes were found to be differentially expressed between *CD55*^high^ and CD55^low^ ASCs (Fig. 4G and Additional file 6: Table S6). Intriguingly, the expression level of several PSP genes that are implemented in anti-inflammation and tissue regeneration is significantly higher in *CD55*^high^ ASCs (the left panel of Fig. 4G). For example, CD55 (a hub gene of the module M1), can be secreted in a soluble form that functions to inhibit complement and immune responses in the fluid phase [17]. Microfibril-associated protein 5 (MFAP5; a hub gene of the module M1), a secreted extracellular matrix component, has been reported to promote angiogenesis and wound healing [18]. Fibrillin-1 (FBN1; a hub gene of module M1), is a secreted extracellular matrix component that regulates MSC differentiation through modulating the bioavailability of TGFβ [19]. METRNL is a novel secreted protein that acts as a neurotrophic factor that promotes neuron regeneration and acts as an adipokine that regulates adipose functions [20]. ANXA2 is a secreted protein that has anti-inflammatory properties and promotes angiogenesis [21]. VEGFA is a canonical growth factor that promotes angiogenesis. In addition, the expression of several genes that promote inflammation was significantly lower in *CD55*^high^ ASCs compared with *CD55*^low^ ASCs, including chemokine genes (e.g., *CXCL14*, *CXCL12*, and *CCL2*) and complement component protein genes (e.g., *C3*, *C7*, and *CFH*; the right panel of Fig. 4G). Together, our data supported the hypothesis that CD55^high^ ASCs have superior treatment efficacy compared with CD55^low^ ASCs.

### In vivo experiments demonstrated that CD55^high^ ASCs had superior treatment efficacy compared with CD55^low^ ASCs

**Fig. 5:**
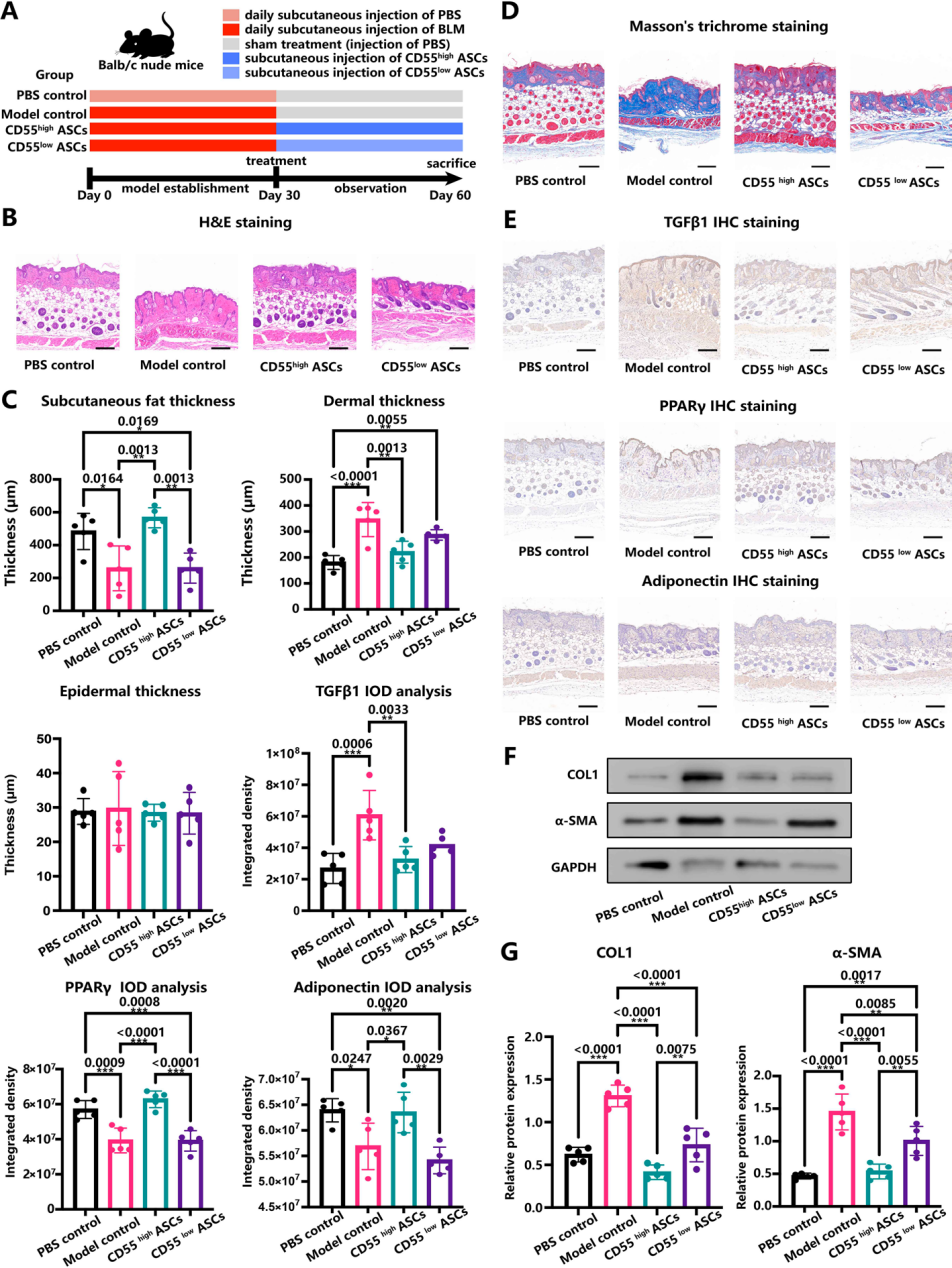
In vivo experiments demonstrated that CD55^high^ ASCs had superior treatment efficacy compared with CD55^low^ ASCs. **A** Schematic showing the experimental design, *n* = 5 for each group of mice. **B** HE staining on skin sections obtained from each group of mice. **C** The subcutaneous fat, dermal, and epidermal thickness of the skin and the protein level of TGFβ1, PPARγ, and Adiponectin for each group. **D** Masson’s trichrome staining showing the degree of collagen deposition and fibrosis for each group. **E** Immunohistochemistry staining showing the protein level of TGFβ1, PPARγ, and Adiponectin for each group. **F** Representative western blotting showing the protein levels of the fibrosis markers COL1 and α-SMA for each group. **G** Quantitative analysis of the western blotting results. In C and G, *: *P* < 0.05; **: *P* < 0.01; ***: *P* < 0.001. One-way ANOVA followed by post hoc tests using the method of Tukey’s HSD (*n* = 5). *BLM* Bleomycin, *IHC* immunohistochemistry, *PBS* phosphate-buffered saline. Scale bar: 200 μm

Next, we proceeded to test the hypothesis in vivo with bleomycin (BLM)-induced skin fibrosis mice models. A total of 20 Balb/c nude mice were assigned to one of four groups: PBS control (daily subcutaneous injection of PBS), model control (daily subcutaneous injection of BLM), CD55^high^ ASC treatment, and CD55^low^ ASC treatment groups (Fig. 5A). The bleomycin-induced mice models exhibited dermal fibrosis and the loss of subcutaneous adipose tissue, which closely resembled the characteristics observed in patients with LoS (Fig. 5B, C). We performed in vivo imaging of DiD-labeled ASCs following subcutaneous injection on the back of bleomycin-induced mouse models and measured the changes in relative fluorescence signal intensity over multiple time points post-injection. By day 7, we observed that 41.6% of the injected cells persisted, whereas by day 28, 10.6% of the cells were still retained. These findings suggest that ASCs can sustain an anti-fibrotic therapeutic effect over the course of our experiment, which spanned 30 days post injection (Fig. S4). Compared with the CD55^low^ ASC treatment, the CD55^high^ ASC treatment group demonstrated significantly greater efficacy in improving subcutaneous fat thickness and decreasing the thickness of the dermis (Fig. 5B, C; Fig. S5). Masson’s trichrome staining demonstrated a notable increase in collagen deposition (a hallmark of skin fibrosis) in the BLM-induced models compared with the PBS control group (Fig. 5D). The collagen deposition was effectively attenuated by both ASC treatment groups. However, the CD55^high^ ASCs had superior treatment efficacy in reducing dermal collagen deposition and skin fibrosis (Fig. 5D). The level of transforming growth factor beta 1 (TGFβ1), a universal driver of fibrosis, was remarkably induced in the skin fibrosis models (Fig. 5E). Only treatment with CD55^high^ ASCs could significantly reduce the level of TGFβ1 (Fig. 5C). Furthermore, the protein level of peroxisome proliferator-activated receptor γ (PPARγ), a key regulator of adipogenesis and lipid metabolism, was significantly reduced in the skin fibrosis models. Treatment with CD55 high ASCs restored this level (Fig. 5C, E). Similarly, the level of adiponectin, an antifibrotic and anti-inflammatory adipokine secreted by subcutaneous fat, was also restored by CD55^high^ ASC treatment (Fig. 5C, E). These findings suggest that treatment with CD55^high^ ASCs may more effectively enhance the functionality of subcutaneous fat in skin fibrosis compared with CD55^low^ ASCs. Western blotting analysis demonstrated a significant reduction in the protein levels of fibrosis markers, namely COL1 and α-SMA, in the skin following ASC treatment (Fig. 5F, G). Notably, CD55^high^ ASCs exhibited a more pronounced effect compared with CD55^low^ ASCs.

### Intercellular communication alterations within the SVF from nonlesional sites of LoS patients compared to healthy donors

**Fig. 6:**
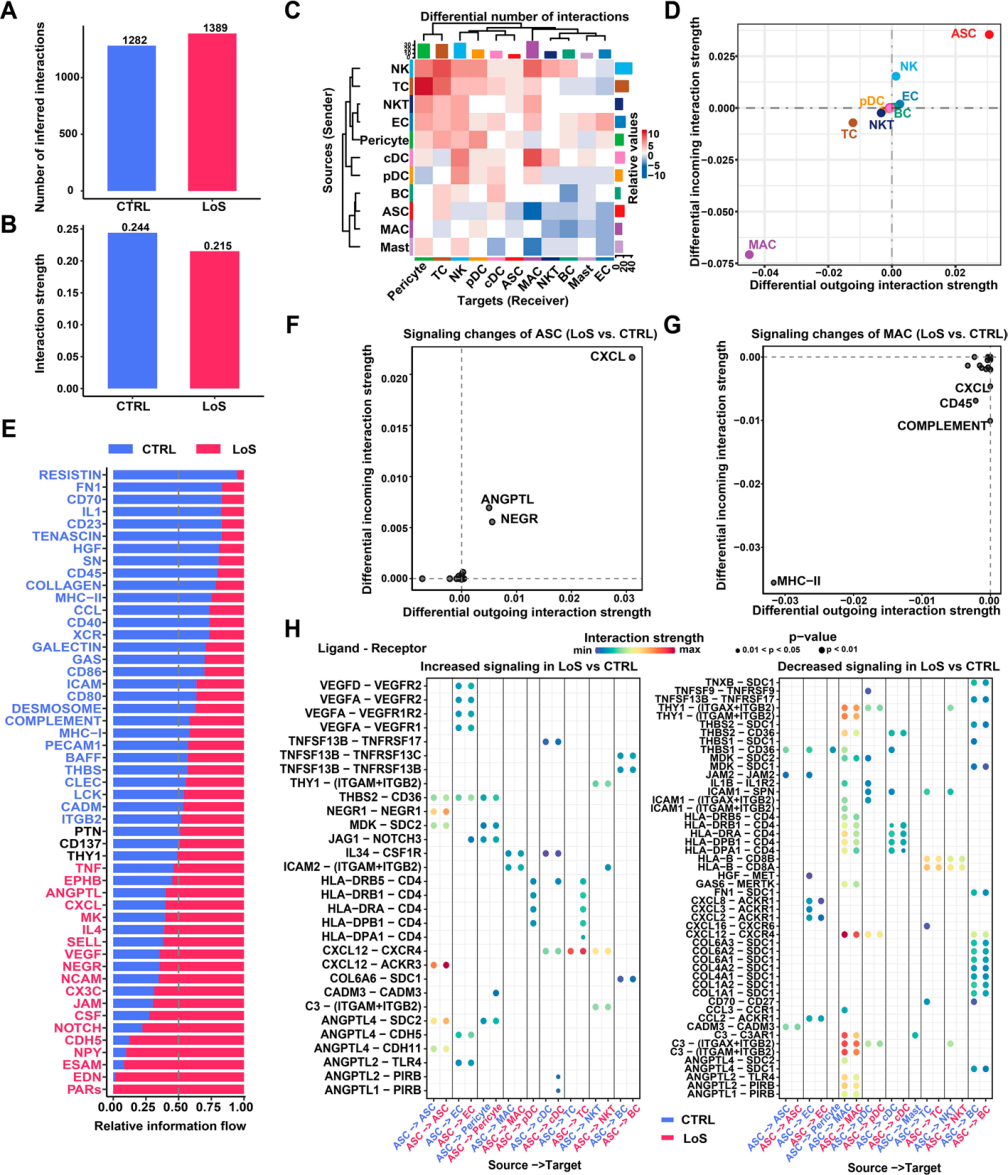
Intercellular communication alterations within the SVF from nonlesional sites of patients with LoS compared with healthy donors. **A** Bar plot showing the total number of ligand–receptor interactions among cell types of each group. **B** Bar plot showing the total interaction strength among cell types of each group. The total interaction strength refers to the accumulated communication probability of all the inferred interactions. **C** Heatmap showing the differential number of interactions among cell types between LoS and CTRL. The bar plot at the top displays the total number of incoming signals for each cell type, while the bar plot on the right shows the total number of outgoing signals for each cell type. **D** Scatter plot showing the differential interaction strength of incoming or outgoing signals for each cell type between groups. **E** Relative information flows for each signaling pathway in each group. The information flow refers to the accumulated communication probability among all cell types. The pathways with increased information flow under the condition of LoS are colored in red, while the pathways with decreased information flow are colored in blue. **F** Signaling pathways with notable changes in ASCs from patients with LoS compared with healthy donors. **G** Signaling pathways with notable changes in macrophages from patients with LoS compared with healthy donors. **H** Ligand‒receptor pairs with increased (left) or decreased (right) interaction strength between ASCs (signal sources) and any of the cell types (signal targets). Only significant interactions are shown (*P*-value < 0.05, the permutation test implemented in CellChat). *ASC* adipose-derived stem cell, *cDC* conventional dendritic cell, *CTRL* control, *EC* endothelial cell, *LoS* localized scleroderma, *MAC* macrophage, *NK* natural killer cell, *NKT* natural killer T cell, *prolif.NKT* proliferative nature killer T cell, *pDC* plasmacytoid dendritic cell

To further understand the mechanisms underlying the functional abnormalities of ASCs, we explored alterations in intercellular communication within the SVF from nonlesional sites of patients with LoS compared with healthy donors using CellChat (Additional file 7: Table S7). Under the condition of LoS, the number of inferred interactions was found to be higher compared with the healthy condition (Fig. 6A). However, the interaction strength was reduced compared with the healthy condition (Fig. 6B). Next, we found that NK and T cells contributed the most to the increased number of interactions in terms of both incoming and outgoing signals (Fig. 6C). Nonetheless, ASCs had a substantial increase in both incoming and outgoing strength, while macrophages exhibited a notable decrease (Fig. 6D). This observation may be attributed to the expansion of ASCs and contraction of macrophages under the condition of LoS. Subsequently, the signaling pathways with increased or decreased information flow (the accumulated communication probability among all cell types) within the SVF under the condition of LoS were identified (Fig. 6E). Furthermore, the signaling pathways with substantial changes in interaction strength for a specific cell type were identified. For example, for ASCs, both the outgoing and incoming strength of the pathways CXCL, ANGPTL, and NEGR increased (Fig. 6F). For macrophages, both the outgoing and incoming strength of the pathways MHC-II decreased (Fig. 6G). Finally, we pinpoint the specific ligand–receptor pairs with increased or decreased interaction strength between ASCs (signal sources) and any cell types (signal targets). Notably, the strength of CXCL12-ACKR3 was enhanced as autocrine signaling of ASCs in LoS (Fig. 6H). The CXCL12/ACKR3 axis has been previously recognized to play a crucial role in various autoimmune diseases [22]. Similarly, enhanced strength of ANGPTL4-SDC2 was also observed as autocrine signaling of ASCs in LoS (Fig. 6H), which may lead to attenuation of Wnt/β-catenin signaling [23], a crucial pathway for MSC self-renewal and differentiation [24].

## Discussion

At present, autologous fat grafting (AFG) is the sole surgical method available for correcting contour deformities caused by LoS. Nevertheless, the retention rate of autologous fat grafts in patients with LoS is typically low. The effectiveness and fat retention rate of AFG could be directly influenced by the cellular quality of the harvested fat from nonlesional sites of patients with LoS, such as the thighs or abdomen. In the present study, we performed a comparative analysis of the single-cell transcriptome of the SVF from nonlesional sites of patients with LoS and healthy donors. Our data supported the hypothesis of inherent abnormalities of ASCs from patients with LoS, and we found that the abnormalities observed in ASCs were partially ascribed to the reduced pool of interstitial progenitor cells (*CD55*^high^) present within the ASC population. Furthermore, we demonstrated that *CD55*^high^ ASCs had superior treatment efficacy compared with *CD55*^low^ ASCs in bleomycin-induced skin fibrosis mice models. To our knowledge, this is the first to demonstrate the potential for improving the efficacy of AFG in the treatment of LoS by restoring the pool of interstitial progenitors within ASCs. These findings have significant implications for the field of translational regenerative medicine.

With the advent of the single-cell omics era, researchers now can unravel cellular heterogeneity within complex tissues, leading to a more profound understanding of the relationship between heterogeneous cellular composition and pathophysiological changes. As an example, Merrick et al. utilized scRNA-seq to identify two subgroups of ASCs in human subcutaneous adipose tissue: multipotent interstitial progenitors (*DPP4*^+^ or *CD55*^high^) and committed preadipocytes (*ICAM1*^+^ or *CD55*^low^), which are restricted to the adipocyte lineage [11]. Further investigation revealed that, in diet-induced obesity mice, the proportion of the multipotent interstitial progenitor subgroup of ASCs in the visceral epididymal white adipose tissue was reduced compared with that from lean controls [11], which implies that the pathological condition of obesity depletes the mesenchymal progenitor pool in adipose tissue. In contrast, another study reported an expansion of the *CD55*^high^ multipotent interstitial progenitors within subcutaneous adipose tissues from patients with cancer-related lymphedema [12]. These results suggest that the proportional alterations of ASC subclusters may be different in response to different pathological environments. Nevertheless, the previous reports highlight the significance of maintaining a proportional balance between multipotent interstitial progenitors and preadipocytes for the homeostasis of adipose tissues. The current study supports this perspective and provides another example of proportional balance changes of ASC subclusters that occur under pathophysiological conditions. We observed a significant reduction in the relative proportion of *CD55*^high^ interstitial progenitor cells in ASCs under the condition of LoS compared with the healthy controls (Fig. 2). The functional abnormalities in ASCs and the low retention rate of autologous fat from LoS may be partially ascribed to the reduced pool of interstitial progenitor cells present within the ASC population. Therefore, developing therapies to restore the proportion of interstitial progenitors or the balance of the two subgroups of ASCs may be a novel therapeutic strategy for LoS.

Autologous fat, SVF, ASCs, and their various derivatives are currently considered promising treatment options for skin fibrosis and subcutaneous adipose tissue atrophy caused by scleroderma [7]. While ASCs have been proven effective in treating skin fibrosis in many studies, negative outcomes should not be overlooked. For instance, a multicenter, randomized clinical trial found that autologous SVF injection did not significantly improve the hand function of SSc patients compared with the placebo [25]. Indeed, one of the major challenges associated with AFG and stem cell-based approaches in the treatment of scleroderma is the instability of treatment effects [26]. Our study revealed a significant difference in the relative proportion of *CD55*^high^ ASCs between patients with LoS and healthy donors (Fig. 2). We also provided in vivo evidence that there was a significant difference in treatment efficacy between *CD55*^high^ ASCs and *CD55*^low^ ASCs (Fig. 5). Mechanistically, *CD55*^high^ ASCs expressed significantly higher levels of secreted protein genes that had the functions related to anti-inflammation and tissue regeneration, and expressed significantly lower levels of secreted protein genes that promotes inflammation, such as chemokine and complement protein genes (Fig. 4). These findings suggest that the variability in the amount and proportion of *CD55*^high^ ASCs among fat samples from different patients may contribute to the instability of treatment efficacy. Therefore, enriching the *CD55*^high^ ASCs of autologous fat through cell sorting before surgery could be a solution to the instability of treatment efficacy. With the safe application of allogeneic ASCs in the treatment of diseases, such as Crohn’s disease [27], isolating *CD55*^high^ ASCs from healthy donors could be a promising approach for the treatment of LoS in the future. This would eliminate the need for multiple traumatic liposuction surgeries and may produce better and more stable treatment effects. However, further pharmacological studies and clinical trials are required before the clinical application of allogeneic *CD55*^high^ ASCs.

Our study uncovered several genes, particularly the secreted protein genes, which have the potential to serve as targets for improving the quality of ASCs. For example, CD55 (decay-accelerating factor), the marker of interstitial progenitors of ASCs, was found to be one of the hub genes of the interstitial progenitor-associated gene module M1 (Fig. 4F), suggesting its significant roles in interstitial progenitors. Moreover, CD55 was found to be significantly downregulated in LoS (Fig. 4G). As a complement inhibitory factor, CD55 promotes the degradation of C3 and C5 convertases, preventing excessive activation of the complement system [28]. As a membrane-associated anchor protein, CD55 has been shown to interact with its receptor CD97, a member of the epidermal growth factor receptor family that is abundantly expressed by macrophages and dendritic cells [29]. CD97 has been demonstrated to inhibit the lipopolysaccharide-induced inflammatory response and TNF-α secretion in macrophages. In addition, CD55 can also be secreted in a soluble form that functions to inhibit complement activation in the fluid phase [17]. On the basis of these findings, we hypothesized that overexpression of CD55 in ASCs could enhance their anti-inflammatory and anti-fibrotic properties. Similarly, *MFAP5*, *METRNL*, and *ANXA2*, which have been implicated in promoting tissue regeneration, were also candidate targets for improving the quality of ASCs in the treatment of LoS.

This study has been limited by the use of immune-compromised mice, which was necessary to avoid immune rejection from xenografts. It should be noted that the immunocompromised mice have altered inflammatory responses and impaired healing mechanisms, which may affect the outcomes of ASC treatments. Consequently, the findings may not translate well to humans. Therefore, clinical studies are essential to validate the treatment’s efficacy in human patients.

## Conclusion

This study revealed that the low retention rate of AFG may be partially ascribed to the reduced pool of interstitial progenitor cells (CD55^high^) present within the ASC population in patients with LoS. This study also demonstrated the potential for improving the efficacy of AFG in the treatment of LoS by restoring the pool of interstitial progenitors within ASCs. Thus, our study has significant implications for the field of translational regenerative medicine.

### Supplementary Information

**Additional file 1: Table S1.** Clinical information of the subjects subjected to scRNA-seq and sequencing quality metrics of the scRNA-seq data.

**Additional file 2: Table S2.** Molecular signature for each cell type or cluster.

**Additional file 3: Table S3.** Differentially expressed genes detected for each major cell type between the SVF from nonlesional sites of patients with LoS and healthy donors.

**Additional file 4: Table S4.** Dysregulated pathways in ASCs from nonlesional sites of patients with LoS detected by gene set enrichment analysis.

**Additional file 5: Table S5.** Gene-coexpression modules identified in ASCs through hdWGCNA.

**Additional file 6: Table S6.** Differentially expressed genes between CD55^high^ and CD55^low^ ASCs.

**Additional file 7: Table S7.** Intercellular communications in the SVF from patients with LoS and healthy donors inferred by Cellchat.

**Additional file 8: Fig. S1.**

## Data Availability

The raw sequence data generated in this study for four LoS samples and one CTRL sample (NSVF11) can be found at Genome Sequence Archive for Human (https://ngdc.cncb.ac.cn/gsa-human/) with the accession number HRA004122. The data for the other four CTRL samples we reanalyzed are available with the accession number HRA000901.

## Abbreviations

AFG: Autologous fat grafting
ASC: Adipose-derived stem cells
DEG: Differentially expressed genes
hdWGCNA: High-dimensional weighted gene coexpression network analysis
LoS: Localized scleroderma
SVF: Stromal vascular fraction
UMAP: Uniform manifold approximation and projection
