# Supplementary material for: Single-cell RNA sequencing identifies inherent abnormalities of adipose-derived stem cells from nonlesional sites of patients with localized scleroderma
Source: Cell Mol Biol Lett. 2024 Aug 30;29:115. doi: 10.1186/s11658-024-00635-0 (PMC11363359; doi:10.1186/s11658-024-00635-0)
Supplement: Supplementary file 8 — Additional file 8: Fig. S1. [file 11658_2024_635_MOESM8_ESM.pdf]

Supplemental Figures

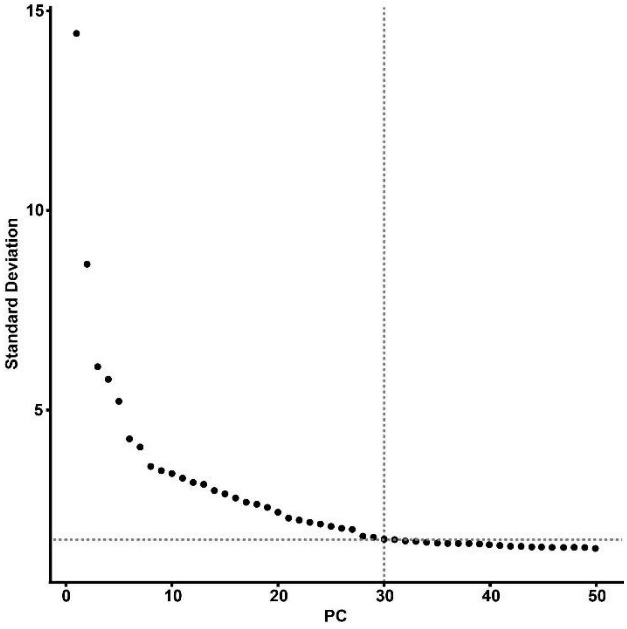

Fig. S1 Elbow plot that illustrates the variance explained by each PC. The first 30 PCs were used to construct the neighborhood graph of the cells. PC: principal component.

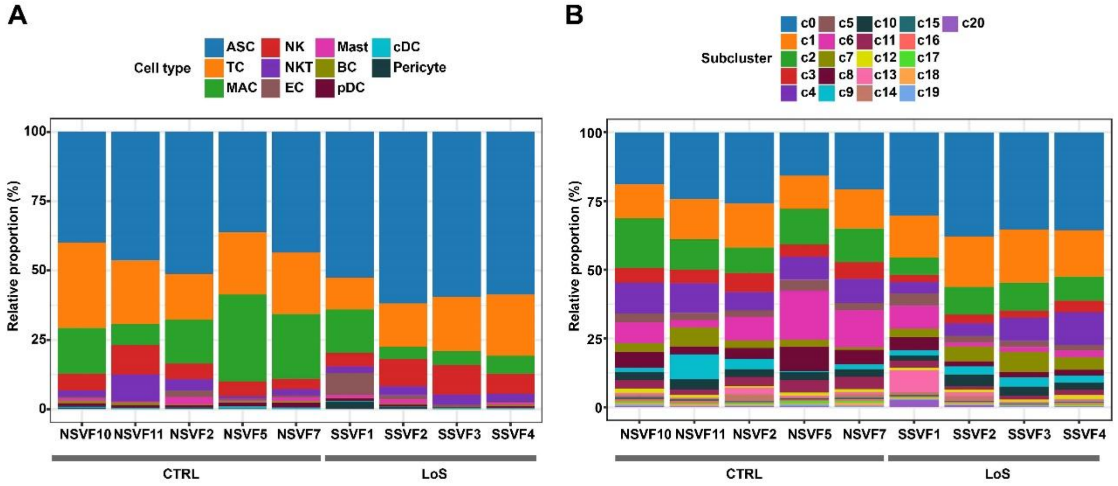

Fig. S2 Stacked bar plots showing the relative proportion of each cell type or subcluster in each sample. **A** Relative proportion of each cell type in each sample. **B** Relative proportion of each subcluster in each sample. ASC: adipose-derived stem cell; cDC: conventional dendritic cell; CTRL: control; EC: endothelial cell; LoS: localized scleroderma; MAC: macrophage; NK: natural killer cell; NKT: natural killer T cell; prolif.NKT: proliferative nature killer T cell; pDC: plasmacytoid dendritic cell.

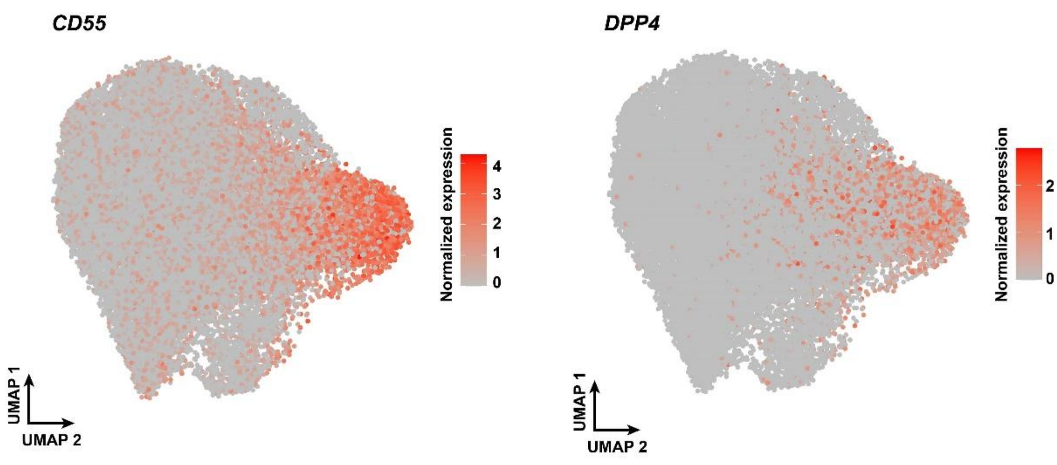

Fig. S3 Dot plots showing the distribution of normalized expression of CD55 and DPP4 in ASCs.

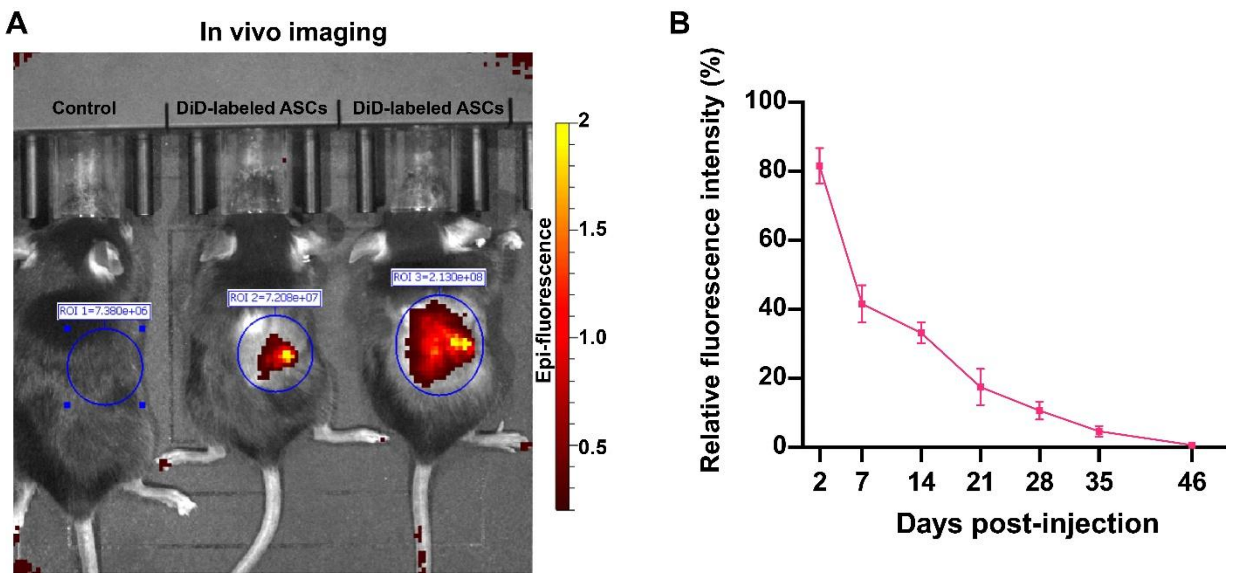

Fig. S4 In vivo imaging of DiD-labeled ASCs following subcutaneous injection on the back of bleomycin-induced mouse models. **A** Representative in vivo image taken on day 14 following subcutaneous injection of DiD-labeled ASCs on the back (5×10^6 cells per animal). ROI: region of interest. **B** Changes in relative fluorescence signal intensity over multiple time points post-injection. n=3.

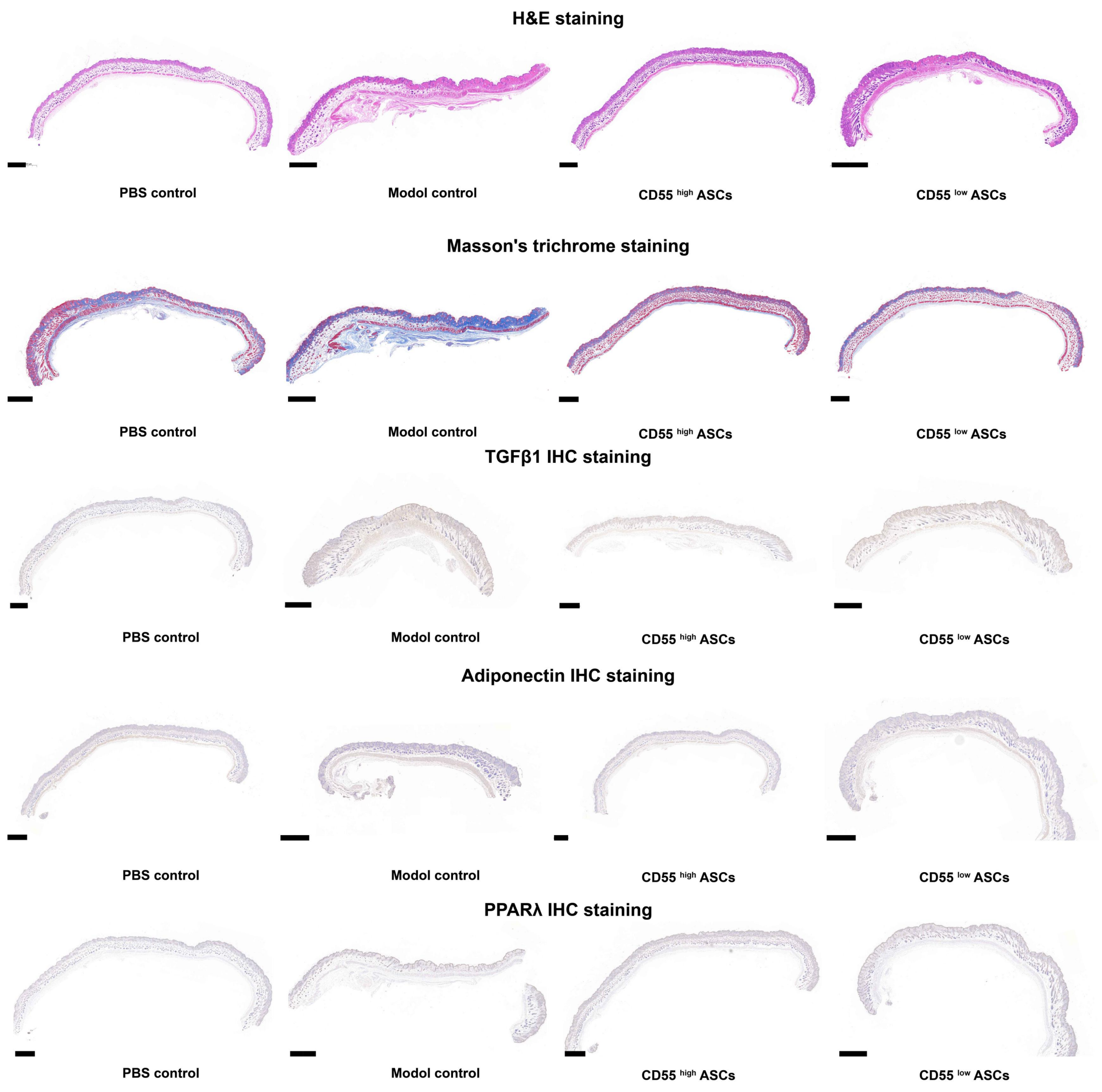

Fig. S5 Representative full tissue section images for each group of mice. Scale bar: 1000 μm
